# Childcare Stress, Burnout, and Intent to Reduce Hours or Leave the Job During the COVID-19 Pandemic Among US Health Care Workers

**DOI:** 10.1001/jamanetworkopen.2022.21776

**Published:** 2022-07-18

**Authors:** Elizabeth M. Harry, Lindsey E. Carlasare, Christine A. Sinsky, Roger L. Brown, Elizabeth Goelz, Nancy Nankivil, Mark Linzer

**Affiliations:** 1 University of Colorado School of Medicine, UCHealth, Aurora; 2 UCHealth, Aurora, Colorado; 3 American Medical Association, Chicago, Illinois; 4 School of Nursing, University of Wisconsin, Madison; 5 School of Medicine and Public Health, University of Wisconsin, Madison; 6 Department of Medicine, Hennepin Healthcare, University of Minnesota, Minneapolis

## Abstract

**Importance:**

Childcare stress (CCS) is high during the COVID-19 pandemic because of remote learning and fear of illness transmission in health care workers (HCWs). Associations between CCS and burnout, intent to reduce (ITR) hours, and intent to leave (ITL) are not known.

**Objective:**

To determine associations between CCS, anxiety and depression, burnout, ITR in 1 year, and ITL in 2 years.

**Design, Setting, and Participants:**

This survey study, Coping with COVID, a brief work-life and wellness survey of US HCWs, was conducted between April and December 2020, assessing CCS, burnout, anxiety, depression, workload, and work intentions. The survey was distributed to clinicians and staff in participating health care organizations with more than 100 physicians. Data were analyzed from October 2021 to May 2022.

**Main Outcomes and Measures:**

The survey asked, “due to…COVID-19, I am experiencing concerns about childcare,” and the presence of CCS was considered as a score of 3 or 4 on a scale from 1, not at all, to 4, a great extent. The survey also asked about fear of exposure or transmission, anxiety, depression, workload, and single-item measures of burnout, ITR, and ITL.

**Results:**

In 208 organizations, 58 408 HCWs (15 766 physicians [26.9%], 11 409 nurses [19.5%], 39 218 women [67.1%], and 33 817 White participants [57.9%]) responded with a median organizational response rate of 32%. CCS was present in 21% (12 197 respondents) of HCWs. CCS was more frequent among racial and ethnic minority individuals and those not identifying race or ethnicity vs White respondents (5028 respondents [25.2%] vs 6356 respondents [18.8%]; *P* < .001; proportional difference, −7.1; 95% CI, −7.8 to −6.3) and among women vs men (8281 respondents [21.1%] vs 2573 respondents [17.9%]; odds ratio [OR], 1.22; 95% CI, 1.17 to 1.29). Those with CCS had 115% greater odds of anxiety or depression (OR, 2.15; 95% CI, 2.04-2.26; *P* < .001), and 80% greater odds of burnout (OR, 1.80; 95% CI, 1.70-1.90; *P* < .001) vs indidivuals without CCS. High CCS was associated with 91% greater odds of ITR (OR, 1.91; 95% CI, 1.76 to 2.08; *P* < .001) and 28% greater odds of ITL (OR, 1.28; 95% CI, 1.17 to 1.40; *P* < .001).

**Conclusions and Relevance:**

In this survey study, CCS was disproportionately described across different subgroups of HCWs and was associated with anxiety, depression, burnout, ITR, and ITL. Addressing CCS may improve HCWs’ quality of life and HCW retention and work participation.

## Key Points

**Question:**

Is high childcare stress (CCS) associated with burnout, intent to reduce clinical hours, and intent to leave the job among US health care workers during the COVID-19 pandemic?

**Findings:**

In this survey study, with 58 408 respondents conducted between April and December 2020, high CCS was associated with 80% greater odds of burnout in all health care workers.

**Meaning:**

These findings suggest there is an association between reporting high CCS and burnout, and programs to reduce CCS may be beneficial for workers and health systems.

## Teaser

This survey study evaluates the association between childcare stress and intent to reduce hours or leave the job during the COVID-19 pandemic among US health care workers.

## Introduction

COVID-19 exacerbated preexisting childcare accessibility issues and disparities.^1^ Before the pandemic, full-time care for 1 infant cost an mean of $21 700 annually in the US.^1^ This cost is greater than one-quarter of the average hospital nurse’s salary, more than one-third of an average medical resident’s salary, and more than two-thirds of a nursing assistant’s salary.^2,3,4^ In addition, many communities do not have childcare available, with childcare desert designations in 3 of 5 rural communities and roughly 60% of Hispanic and Latinx population areas.^5^ Health care workers (HCWs) have the added difficulty of trying to find care outside typical hours such as nights and weekends, with only 8% of the center-based care providing nonstandard coverage.^5^ COVID-19 exacerbated many of these preexisting issues via school closures and day care centers losing nearly 70% of their daily attendance within 1 week in mid-March 2020.^5^

Besides childcare disparities, the pandemic exacerbated mental health concerns, more so for female HCWs, leading to a disparity not previously present. Female HCWs have been found to have higher odds of experiencing depression, anxiety, stress, and insomnia during the pandemic after adjusting for cofounders.^6,7,8^ Coping with added mental health concerns, female HCWs also bore a larger burden of home challenges during the pandemic, including childcare, schooling, and household tasks.^9^ The combination of the extra duties and psychological stress affected female HCWs’ professional advancement. Publications with women as first authors have declined since onset of the pandemic.^10^ Notably, institutional involvement in providing childcare has been shown to decrease childcare stress (CCS).^11^

This increase in stress for HCWs during COVID-19 correlates with an increased intent to leave (ITL) their role or intent to reduce (ITR) clinical hours in the next year and a previously documented 25% to 35% follow-through rate on ITL.^7,8,12^ Since the start of the pandemic, 1 in 5 HCWs has quit their job according to a poll conducted in September 2021.^13^ Given the unique CCS generated during the pandemic and the increase in those leaving or with ITL and ITR, we sought to determine the prevalence and associations of CCS among HCWs during the COVID-19 pandemic.^12^

## Methods

### Study Design

This survey study followed the American Association for Public Opinion Research (AAPOR) reporting guideline. The Hennepin Healthcare institutional review board deemed this study a quality improvement and program evaluation project exempt from research requirements and the need for informed consent in accordance with 45 CFR §46.

The Coping with COVID survey (eAppendix in the Supplement), the source of the data used in this evaluation, has been described elsewhere.^14^ Briefly, we surveyed clinicians and staff in health care organizations with more than 100 physicians. Participation was through invitation or word of mouth. This study includes data collected between April and December 2020. The final sample size for the burnout models was reduced by approximately 38% because of lack of collecting information regarding ITR and ITL in many organizations. Missing data analysis showed that the other missingness was missing at random. No data imputation was done.

### Survey Measures

The Coping with COVID survey (eAppendix in the Supplement), adapted in part from existing measures,^15^ is a 14-item survey plus several demographic items (race and ethnicity [Asian or Pacific Islander, Black or African American, Hispanic or Latino, Native American or American Indian, White, multiracial], gender, years in practice, outpatient vs inpatient practice environment, and work role). Race and ethnicity were analyzed in this study because it was plausible that existing disparities in child care were exacerbated during COVID-19. The survey includes a single-item stress measure, questions about fear of exposure or transmission of the virus, anxiety and depression symptoms attributed to COVID-19, and work overload, all measured with 4-point Likert scales. CCS was assessed with a single item asking, “Due to the impact of COVID-19, I am experiencing concerns about childcare,” with responses ranging from not at all to somewhat, moderately, and to a great extent (scored 1-4, with a higher score indicating higher CCS). Single-item questions adapted from the Minimizing Error Maximizing Outcome study^16^ assessed ITR in 1 year or leaving the job within 2 years on 5-point scales from unlikely to definite, with scores of 3, 4, or 5 representing ITR or ITL. The survey’s single item assessing burnout, validated against the Maslach Burnout Inventory’s emotional exhaustion subscale, was scored from no burnout (1) through highly burned out (5). Those indicating 3, 4, or 5 (selections including the word “burnout”) were considered burned out. As in prior Coping with COVID publications, for other questions with response choices ranging from 1 (not at all) to 4 (very high), responses of 3 or 4 were considered high. Construct validity for aspects of the Coping with COVID survey has been previously described.^14,17^

### Statistical Analysis

Descriptive statistics were used to describe the sample; multivariable comparisons were performed using 2-tailed χ^2^ and *t* tests with significance set at *P* < .05. Two-level (respondent within organization) logistic risk regression models were performed to assess correlates of burnout, anxiety, depression, ITR, and ITL. Adjusted odds ratio (AOR), adjusted risk ratio, and adjusted risk difference are presented; adjusted risk ratio is the multiplicative increase in risk resulting from exposure, conditional on covariates, whereas adjusted risk difference represents the difference between the adjusted risk of those exposed vs those unexposed.^18^ Analyses were completed in Stata/SE, version 17.0 (StataCorp). Data were analyzed from October 2021 to May 2022.

## Results

### Demographics

Of 58 408 respondents at 208 health care organizations (Table 1), 33 817 (58%) were White and 39 218 (67%) were female (median response rate, 32%). There were 15 766 physicians (27%), including 4418 (8%) family physicians, 3194 (6%) in general internal medicine, 3001 (5%) in pediatrics, and 1328 (2%) in hospital medicine (Table 2). There were 11 409 (20%) nurses and 5415 (9%) administrative staff. Other role types are listed in Table 1, along with years in practice.

**Table 1.** Characteristics of Participants in the Coping With COVID Study

Characteristic	Participants, No. (%) (N = 58 408)
Race and ethnicity	
Asian or Pacific Islander	4803 (8.22)
Black or African American	3462 (5.93)
Hispanic or Latino	3222 (5.52)
Native American or American Indian	119 (0.2)
White	33 817 (57.9)
Missing	5191 (8.89)
Multiracial	947 (1.62)
Prefer not to answer	6847 (11.72)
Gender	
Missing	4 (0.01)
Male	14 377 (24.61)
Female	39 218 (67.14)
Nonbinary or third gender	158 (0.27)
Prefer not to answer	4651 (7.96)
Years in practice	
Missing	21 (0.04)
Not available	6788 (11.62)
1-5	12 108 (20.73)
6-10	9128 (15.63)
11-15	7534 (12.90)
16-20	6101 (10.45)
>20	16 728 (28.64)
Role	
Missing	46 (0.08)
Physician	15 766 (26.99)
Advanced practice practitioner	4409 (7.55)
Nurse	11 409 (19.53)
Pharmacist	790 (1.35)
Nursing assistant	1136 (1.94)
Housekeeping	235 (0.40)
Respiratory therapist	339 (0.58)
Physical therapist	869 (1.49)
Occupational therapist	240 (0.41)
Speech therapist	148 (0.25)
Administrative	5415 (9.27)
Medical assistant	1249 (2.14)
Receptionist or scheduler	1486 (2.54)
Resident or fellow	2346 (4.02)
Social worker	1 (0.01)
Laboratory or radiology technician	851 (1.46)
Finance	1103 (1.89)
Food service	191 (0.33)
Information technology support	803 (1.37)
Researcher (without clinical role)	569 (0.97)
Laboratory staff	718 (1.23)
Other	8289 (14.19)
Setting	
Missing	53 229 (91.13)
Inpatient	1900 (3.25)
Outpatient	3279 (5.61)

**Table 2.** Multivariable Comparisons of Childcare Stress

Variable	Participants, No. (%)			OR (95% CI)
	Missing data^a^	No CCS	CCS	
Race and ethnicity				
Asian or Pacific Islander	2 (0.04)	3596 (74.87)	1205 (25.09)	1.44 (1.34-1.55)
Black or African American	2 (0.06)	2606 (75.27)	854 (24.67)	1.41 (1.30-1.53)
Hispanic or Latino	3 (0.09)	2371 (73.59)	848 (26.32)	1.54 (1.42-1.67)
Native American or American Indian	0	94 (78.99)	25 (21.01)	1.14 (0.73-1.78)
White	62 (0.18)	27 399 (81.02)	6356 (18.80)	1 [Reference]
Multiracial	0	739 (78.04)	208 (21.96)	1.21 (1.03-1.41)
Prefer not to answer	6 (0.09)	4953 (72.34)	1888 (27.57)	1.64 (1.54-1.74)
Missing	2 (0.04)	4376 (84.30)	813 (15.66)	NA
Gender				
Male	10 (0.07)	11 794 (82.03)	2573 (17.90)	1 [Reference]
Female	60 (0.15)	30 877 (78.73)	8281 (21.12)	1.22 (1.17-1.29)
Nonbinary or third gender	0	106 (67.09)	52 (32.91)	2.24 (1.60-3.14)
Prefer not to answer	6 (0.13)	3354 (72.11)	1291 (27.76)	1.76 (1.63-1.90)
Missing	1 (25)	3 (75.00)	0	NA
Specialty				
Family medicine	3 (0.07)	3424 (77.50)	991 (22.43)	1 [Reference]
Allergy and immunology	0	101 (69.18)	45 (30.82)	1.53 (1.07-2.20)
Anesthesiology	0	966 (76.79)	292 (23.21)	1.04 (0.90-1.21)
Cardiac or thoracic surgery	0	408 (78.01)	115 (21.99)	0.97 (0.78-1.21)
Cardiovascular diseases	2 (0.13)	1183 (78.29)	326 (21.58)	0.95 (0.82-1.09)
Dentistry or oral surgery	0	309 (79.84)	78 (20.16)	0.87 (0.67-1.12)
Dermatology	0	172 (68.80)	78 (31.20)	1.56 (1.18-2.06)
Emergency medicine	3 (0.11)	2112 (78.51)	575 (21.38)	0.94 (0.83-1.05)
Gastroenterology	1 (0.18)	424 (76.67)	128 (23.15)	1.04 (0.84-1.28)
General practice	1 (0.11)	714 (76.53)	218 (23.37)	1.05 (0.89-1.24)
Hematology or oncology	1 (0.18)	438 (77.52)	126 (22.30)	0.99 (0.80-1.22)
Hospitalist	0	998 (75.15)	330 (24.85)	1.14 (0.99-1.31)
Infectious disease	2 (0.42)	359 (75.58)	114 (24.00)	1.09 (0.87-1.37)
Internal medicine, general medicine, or primary care	1 (0.03)	2514 (78.71)	679 (21.26)	0.93 (0.83-1.04)
Nephrology	0	288 (76.39)	89 (23.61)	1.06 (0.83-1.36)
Neurological surgery	0	219 (80.22)	54 (19.78)	0.85 (0.62-1.15)
Neurology	2 (0.24)	631 (76.02)	197 (23.73)	1.07 (0.90-1.28)
Obstetrics and gynecology	0	1702 (78.07)	478 (21.93)	0.97 (0.85-1.09)
Oncology	0	791 (77.40)	231 (22.60)	1.00 (0.85-1.18)
Ophthalmology	1 (0.21)	349 (74.41)	119 (25.37)	1.17 (0.94-1.46)
Orthopedic surgery	2 (0.15)	1046 (79.42)	269 (20.43)	0.88 (0.76-1.03)
Otolaryngology	0	303 (78.70)	82 (21.30)	0.93 (0.72-1.20)
Palliative care	1 (0.33)	243 (79.93)	60 (19.74)	0.85 (0.63-1.14)
Pathology	0	430 (80.37)	105 (19.63)	0.84 (0.67-1.05)
Pediatrics	1 (0.03)	2300 (76.64)	700 (23.33)	1.05 (0.94-1.17)
Physical and occupational therapy	4 (0.38)	803 (77.29)	232 (22.33)	0.99 (0.84-1.17)
Physical medicine and rehabilitation	1 (0.19)	423 (79.07)	111 (20.75)	0.90 (0.72-1.13)
Plastic surgery	0	98 (83.76)	19 (16.24)	0.66 (0.40-1.10)
Podiatry	0	132 (80.49)	32 (19.51)	0.83 (0.56-1.24)
Psychiatry	0	1682 (77.33)	493 (22.67)	1.01 (0.89-1.14)
Pulmonary disease	2 (0.41)	397 (81.19)	90 (18.40)	0.78 (0.61-0.99)
Radiation oncology	0	147 (77.78)	42 (22.22)	0.98 (0.69-1.40)
Radiology	0	1247 (79.17)	328 (20.83)	0.90 (0.78-1.04)
Rheumatology	1 (0.54)	139 (74.73)	46 (24.73)	1.14 (0.81-1.60)
Surgery, general	2 (0.12)	1328 (80.34)	323 (19.54)	0.84 (0.73-0.96)
Urological surgery	0	225 (76.79)	68 (23.21)	1.04 (0.78-1.38)
Vascular surgery	0	128 (83.66)	25 (16.34)	0.67 (0.43-1.04)
Other specialty				
Surgery-related	2 (0.20)	798 (80.12)	196 (19.68)	0.84 (0.71-1.00)
Nonsurgery related	5 (0.18)	2238 (79.59)	569 (20.23)	0.87 (0.78-0.98)
Critical care medicine	1 (0.06)	1232 (77.73)	352 (22.21)	0.98 (0.86-1.13)
Missing	38 (0.24)	12 693 (8.177)	2792 (17.99)	NA
Years in practice				
1-5	17 (0.14)	9530 (78.71)	2561 (21.15)	1 [Reference]
6-10	4 (0.04)	6044 (66.21)	3080 (33.74)	1.89 (1.78-2.01)
11-15	4 (0.05)	4944 (65.62)	2586 (34.32)	1.94 (1.82-2.07)
16-20	4 (0.07)	4665 (76.46)	1432 (23.47)	1.14 (1.06-1.22)
>20	38 (0.23)	15 246 (91.14)	1444 (8.63)	0.35 (0.32-0.37)
Missing	3 (14.29)	13 (61.90)	5 (23.81)	NA
NA	7 (0.10)	5692 (83.85)	1089 (16.04)	NA
Role				
Physician	4 (0.03)	12 191 (77.32)	3571 (22.64)	1 [Reference]
Advanced practice practitioner	5 (0.11)	3329 (75.50)	1075 (24.38)	1.10 (1.01-1.19)
Nurse	13 (0.11)	9146 (80.16)	2250 (19.72)	0.83 (0.79-0.89)
Pharmacist	2 (0.25)	586 (74.18)	202 (25.57)	1.17 (0.99-1.38)
Nursing assistant	0	847 (74.56)	289 (25.44)	1.16 (1.01-1.33)
Housekeeping	0	207 (88.09)	28 (11.91)	0.46 (0.31-0.68)
Respiratory therapist	1 (0.29)	281 (82.89)	57 (16.81)	0.69 (0.51-0.92)
Physical therapist	5 (0.58)	669 (76.99)	195 (22.44)	0.99 (0.84-1.17)
Occupational therapist	0	188 (78.33)	52 (21.67)	0.94 (0.69-1.28)
Speech therapist	0	104 (70.27)	44 (29.73)	1.44 (1.01-2.05)
Administrative	11 (0.20)	4319 (79.76)	1085 (20.04)	0.85 (0.79-0.92)
Medical assistant	3 (0.24)	910 (72.86)	336 (26.90)	1.26 (1.10-1.43)
Receptionist/scheduler	6 (0.40)	1203 (80.96)	277 (18.64)	0.78 (0.68-0.90)
Resident or fellow	2 (0.09)	1857 (79.16)	487 (20.76)	0.89 (0.80-0.99)
Social worker	0	1 (100)	0	NA
Laboratory or radiograph technician	2 (0.24)	683 (80.26)	166 (19.51)	0.82 (0.69-0.98)
Finance	1 (0.09)	859 (77.88)	243 (22.03)	0.96 (0.83-1.11)
Food service	0	158 (82.72)	33 (17.28)	0.71 (0.48-1.04)
Information technology support	2 (0.25)	646 (80.45)	155 (19.30)	0.81 (0.68-0.97)
Researcher (without clinical role)	0	442 (77.68)	127 (22.32)	0.98 (0.80-1.19)
Laboratory staff	2 (0.28)	585 (81.48)	131 (18.25)	0.76 (0.63-0.92)
Other (please specify)	15 (0.18)	6886 (83.07)	1388 (16.75)	0.68 (0.64-0.73)
Missing	3 (6.52)	37 (80.43)	6 (13.04)	NA

Abbreviations: CCS, childcare stress; NA, not applicable; OR, odds ratio.

^a^ Missing data were not included in comparisons; 95% CIs were not adjusted for multiple testing.

CCS was experienced by approximately 21% (12 197) of all workers. As shown in Table 2, CCS was more frequently noted among racial and ethnic minority groups vs White individuals (5028 [25.2%] vs 6356 respondents [18.8%]; *P* < .001) and among women vs men (8281 respondents [21%] vs 2573 respondents [18%]; OR, 1.22; 95% CI, 1.17-1.29; *P* < .001). Racial and ethnic minority individuals had 40 to 50% greater odds of reporting CCS than white respondents and women had 22% greater odds of reporting CCS than men. CCS was particularly frequent among nonbinary respondents (52 of 158 respondents [33%] with CCS; OR, 2.24; 95% CI, 1.60-3.14; *P* < .001) and those preferring not to report their gender identity (1291 respondents [28%] with CCS; OR, 1.76; 95% CI, 1.63-1.90; *P* < .001). Most specialties had comparable prevalence of CCS to family medicine (991 respondents [22%]); 2 smaller specialties with high CCS included allergy (45 respondents [30.8%]) and dermatology (78 respondents [31.2%]). CCS was most often seen in those in practice 6 to 10 years (OR, 1.89; 95% CI, 1.78-2.01; *P* < .001) and 11-15 years (OR, 1.94; 95% CI, 1.82-2.07; *P* < .001) vs those in practice 1 to 5 years. Within role types, CCS was most often noted among medical assistants (336 respondents [26.9%]), nursing assistants (289 respondents [25.4%]), speech therapists (44 respondents [29.7%]), and pharmacists (202 respondents [25.6%]) vs 3571 physicians (23%).

The Figure shows how CCS was associated with burnout and anxiety and depression in all HCWs and in physicians, stratified by gender. The prevalence of burnout was substantially higher among those with high CCS. In all HCWs, 39% of men with low CCS had burnout, whereas 53% of men with high CCS had burnout, a relative increase of 35%. For female HCWs, the 49% with low CCS had burnout vs 63% of those with high CCS, a 28% increase; these numbers were comparable in the physician-only group. For anxiety and depressive symptoms from COVID-19, the increases were more striking; among male HCW with low CCS, 23% reported anxiety or depression vs 43% of men with high CCS (a relative increase of 86%), and for female HCWs, the numbers were 33% vs 50%, a 51% increase. These numbers were comparable for the all-physician analysis.

**Figure.**
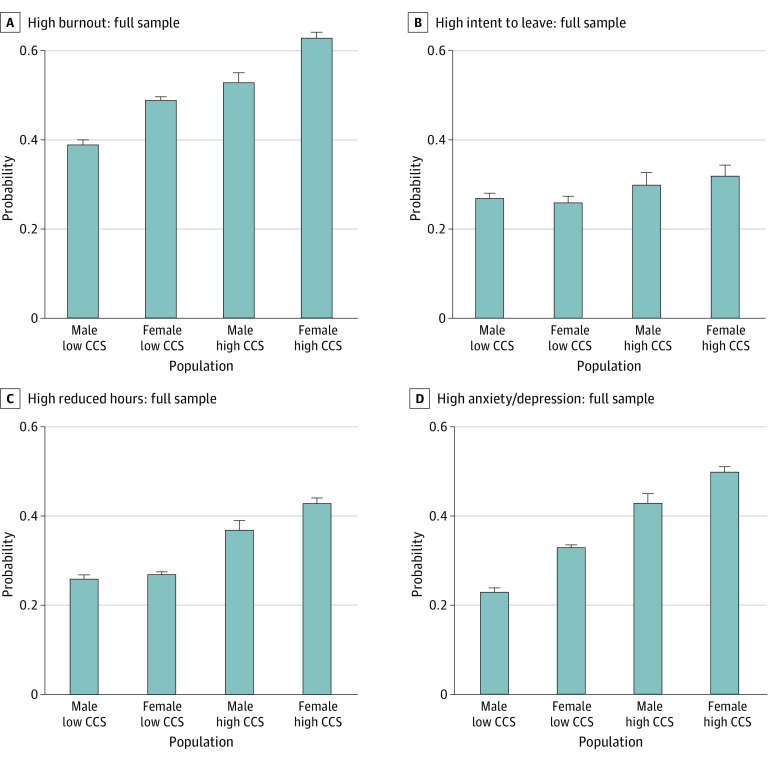
Burnout, Intent to Leave, Intent to Reduce Hours, and Anxiety and Depression by Childcare Stress (CCS) Level and Gender

### Burnout

Logistic regression results for all workers (Table 3, model 1) controlling for years in practice, specialty, and role showed women had about 50% greater odds of reporting burnout than men (AOR, 1.49; 95% CI, 1.41-1.57; *P* < .001). All workers experiencing CCS had 80% greater odds of burnout than those with low CCS (AOR, 1.80; 95% CI, 1.70-1.90; *P* < .001). When the model was constructed just on physicians (Table 3, model 2), findings were similar. Burnout models expanded to include the interaction of gender and CCS are found in eTable 1 in the Supplement (models 2 and 4). No significant interaction was discovered. Pseudo *R*^2^ was estimated by the method of McKelvey et al^19^ for percentage variance explained in latent burnout because of CCS, and was fairly low at 5% to 6%, suggesting unmeasured variables, including work conditions, may comprise a larger share of factors associated with increased risk of burnout.

**Table 3.** Multivariate (Logit) Models of Factors Associated WIth Burnout

Variable	Model 1: full sample (n = 35 998)^a^				Model 2: physicians only (n = 12 888)^b^			
	AOR (95% CI)	*P* value	ARR (95% CI)	ARD, % (95% CI)	AOR (95% CI)	*P* value	ARR (95% CI)	ARD, % (95% CI)
Female gender	1.49 (1.41-1.57)	<.001	1.22 (1.18-1.25)	9.6 (8.3-10.8)	1.54 (1.43-1.67)	<.001	1.25 (1.20-1.30)	10.4 (8.6-12.2)
High childcare stress risk	1.80 (1.70-1.90)	<.001	1.30 (1.27-1.33)	14.2 (12.9-15.4)	1.92 (1.75-2.10)	<.001	1.37 (1.32-1.43)	15.8 (13.6-17.9)
Intercept	0.39 (0.26-0.58)	<.001	NA	NA	0.28 (0.14-0.56)	<.001	NA	NA

Abbreviations: AOR, adjusted odds ratio; ARD, adjusted risk difference; ARR, adjusted risk ratio; NA, not applicable.

^a^ Model 1 was adjusted for specialty, years in practice, and role (McKelvey and Zavoina pseudo *R*^2^ = 0.053).

^b^ Model 2 was adjusted for specialty and years in practice (McKelvey and Zavoina pseudo *R*^2^ = 0.060).

### Intent to Reduce

Logistic regressions for all HCWs (Table 4, model 1) controlling for years in practice, specialty, and role showed that women had significantly greater odds of reporting ITR than men (AOR, 1.11; 95% CI, 1.02 to 1.20; *P* < .001). All workers experiencing CCS had 91% greater odds of ITR than those with low CCS (AOR, 1.91; 95% CI, 1.76 to 2.08; *P* < .001). When the model was constructed for physicians (Table 4, model 2), no significant difference was detected by gender (AOR, 1.07; 95% CI, 0.97-1.19; *P* = .15). However, in all physicians, those experiencing CCS had 92% greater odds of reducing hours than those with low CCS (AOR, 1.92; 95% CI, 1.71-2.15; *P* < .001). ITR models were expanded to include the interaction of gender and CCS (eTable 2 in the Supplement, models 2 and 4). An interaction was discovered for both the all-HCW sample (AOR, 1.18; 95% CI, 1.00-1.40; *P* = .05), and physician-only sample (AOR, 1.26; 95% CI, 1.01-1.56; *P* = .03), showing a greater increase in odds of ITR in women vs men associated with CCS; this association was significant in physicians. A small pseudo *R*^2^ was estimated for these models.

**Table 4.** Multivariate (Logit) Models of Likelihood to Reduce Hours

Variable	Model 1: full sample (n = 15 807)^a^				Model 2: physicians only (n = 8722)^b^			
	AOR (95% CI)	*P* value	ARR (95% CI)	ARD, % (95% CI)	AOR (95% CI)	*P* value	ARR (95% CI)	ARD, % (95% CI)
Female gender	1.11 (1.02 to 1.20)	<.001	1.07 (1.01 to 1.13)	2.1 (0.5 to 3.8)	1.07 (0.97 to 1.19)	.15	1.05 (0.98 to 1.12)	1.5 (−0.6 to 3.6)
High childcare stress risk	1.91 (1.76 to 2.08)	<.001	1.52 (1.44 to 1.60)	14.1 (12.2 to 16.0)	1.92 (1.71 to 2.15)	<.001	1.51 (1.41 to 1.62)	14.1 (11.6 to 16.7)
Intercept	0.17 (0.08 to 0.38)	<.001	NA	NA	0.22 (0.06 to 0.72)	<.001	NA	NA

Abbreviations: AOR, adjusted odds ratio; ARD, adjusted risk difference; ARR, adjusted risk ratio; NA, not applicable.

^a^ Model 1 was adjusted for specialty, years in practice, and role (McKelvey and Zavoina pseudo *R*^2^ = 0.059).

^b^ Model 2 was adjusted for specialty and years in practice (McKelvey and Zavoina pseudo *R*^2^ = 0.051).

### Intent to Leave

Logistic regressions for all workers (eTable 3 in the Supplement, model 1), controlling for years in practice, specialty, and role, showed that women had similar odds of ITL vs men (OR, 0.97; 95% CI, 0.89-1.06; *P* = .64). All workers experiencing CCS had 28% greater odds of ITL vs those with low CCS (OR, 1.28; 95% CI, 1.17-1.40; *P* < .001). When the model was constructed just on physicians (eTable 3 in the Supplement, model 3), similar findings were noticed. When ITL models were expanded to include the interaction of gender and CCS (eTable 3 in the Supplement, models 2 and 4), no differential effect was discovered. Similarly, a small pseudo *R*^2^ was estimated.

### Anxiety and Depression

Logistic regression results for all workers (eTable 4 in the Supplement, model 1), controlling for years in practice, specialty, and role, showed that women had significantly greater odds of experiencing anxiety and depression vs men (AOR, 1.56; 95% CI, 1.47-1.65; *P* < .001. The adjusted risk differences and adjusted risk ratios are also shown in eTable 4 in the Supplement. All workers experiencing CCS had 115% greater odds of anxiety and depression vs those with low CCS (AOR, 2.15; 95% CI, 2.04-2.26; *P* < .001). When the model was constructed just on physicians (eTable 4 in the Supplement, model 3), a significant difference was observed with greater odds of anxiety and depression in women vs men (AOR, 1.55; 95% CI, 1.51-1.59; *P* < .001). Physicians experiencing CCS had 111% greater odds of anxiety and depression vs those with low CCS (AOR, 2.11; 95% CI, 1.92-2.31; *P* < .001). The anxiety and depression models, expanded to include the interaction of gender and CCS (eTable 4 in the Supplement, models 2 and 4), demonstrated a significant differential effect, with men having a greater odds of increase in anxiety and depression at comparable levels of CCS. A small pseudo *R*^2^ was estimated for these models.

## Discussion

In this survey study, we found that CCS during the pandemic was prevalent among HCWs, more so in women vs men, racial and ethnic minority groups vs White individuals, and those with 6 to 15 years of practice vs those with 1 to 5 years. Racial and ethnic minority individuals had 40% to 50% greater odds of reporting CCS than White respondents and women had 22% greater odds of reporting CCS than men. These findings are discussed further in the work by Prasad et al,^18^ who reported that those in early to mid-career at 6 to 15 years of practice had 90% greater odds of reporting CCS than those beginning their career. It is important to recognize the vulnerability of these populations to CCS, particularly given the association between CCS and other concerning features such as burnout, anxiety and depression, and ITL or ITR.

To our knowledge, this is the first study in such a large population of diverse HCWs to report the prevalence of CCS during the pandemic on a national scale and to link those concerns with burnout, mental health, and work intentions. All populations experiencing higher CCS had higher rates of burnout. Multivariable comparisons highlight challenges CCS pose for racial and ethnic minority individuals and those who preferred not to answer the race and ethnicity question. CCS was known to be higher in racial and ethnic minority communities before the pandemic and continued to increase with the onset of the pandemic.^20^ Recovery based on racial equity needs to include collecting data, involving racial and ethnic minority communitiesin the process, and increasing access to childcare going forward.^20^ Without these efforts, individuals from minoritized groups will probably experience reduced participation in the workforce. Thus, reasons for CCS in racial and ethnic minority groups should be addressed by health care organizations and explored in future scholarly studies.

CCS findings have practical and financial implications, ranging from the increased risk of self-reported medical error to turnover and other costs associated with burnout. HCWs’ occupational stress (ie, burnout) and mental health are of major national concern, and physician turnover and reduced clinical effort due to burnout are estimated to cost $4.6 billion annually, with nursing burnout–related turnover adding $14 billion annually.^17,21,22,23^ With HCW stress exacerbation during COVID-19, attention to childcare needs and CCS are strategies health care organizations can use to address HCW well-being.

In regression analyses controlling for years in practice, specialty, and role, women had approximately 50% greater odds of experiencing burnout than male colleagues, independent of CCS. Workers experiencing high CCS had 80% to 90% greater odds of experiencing burnout than workers reporting low CCS. Multivariate models (eTables 1-4 in the Supplement) found that men experiencing CCS had significantly greater odds than women in reporting anxiety and depression, whereas women experiencing CCS had greater odds of an ITR than men (Figure). Thus, although there are gender-related findings, an overarching result is that CCS appears to affect both female and male HCWs and concerns large numbers of HCWs, with significant findings seen in both all-HCW and physician-only analyses. Attending to CCS may help lower burnout rates for women, who historically have higher burnout rates than men.^24^ Given the increased burden women face at home, removing barriers for men in their participation in home duties is critical. Recognizing that men who are experiencing high CCS have strong odds of reporting anxiety and depression is important in discussing ways to support removing CCS burden from both male and female HCWs.

What can be done? We propose a more intentional approach in the health care workplace to assessing and addressing childcare concerns when worker assignments are made. Workplaces that can accommodate change on short notice, provide on-site care for ill children or on-site schools, and are aware of worker concerns about their children will be better positioned to show workers they are a caring environment, one that, we hope, workers would be more likely to remain with rather than leaving for shift work in other settings, a scenario that is currently occurring in large numbers. As Rachel et al^11^ described, work-affiliated childcare reduces CCS and would be a reasonable strategy to mitigate the impact of childcare stress on ITL or ITR.

### Limitations and Strengths

This study has several limitations, including its self-reported nature, the uncertain association between ITR or ITL and acting on those intentions, the confounding associations between burnout contributing to ITL or ITR vs CCS contributing to burnout (which contributes to turnover), the potential differences in responders and nonresponders, and the chaotic (pandemic-related) circumstance during which the survey was conducted. This survey is completed by organizations who opt in. This choice may suggest a higher engagement in well-being initiatives. It is possible that this underestimates CCS in a generic organization. Response rates are less than optimal; however, this study was conducted during a stressful time of the pandemic, and some organizations were still enrolling participants when the data set was closed. Rates obtained still exceeded those in national physician surveys conducted before the pandemic. Additionally, low response rates do not ensure lack of a representative sample.^25^

Because of the cross-sectional nature of the study, we can in no way identify a relationship between CCS and burnout, nor can we relate the findings to CCS during usual times. The pandemic did, however, uncover institutional barriers that marginalize certain populations; thus, it is possible CCS was a factor associated with burnout and retention before the pandemic and will continue to be relevant going forward. More information about the respondents, such as partnership status, number and age of children, and partner occupation would provide more insight into these data. The differences found between high CCS and low CCS without knowledge of these additional variables, however, suggest even stronger differences would have been seen had we been able to exclude those without children. Additionally, several survey items were not validated (eg, anxiety and depression) against standard metrics, and thus may be difficult to interpret.

Strengths include the validated survey, the data for a diverse all-HCW sample with many roles represented, the size of the sample, and the timing of capturing data during a vulnerable period. Further studies evaluating CCS, burnout, anxiety, and ITR or ITL before and after childcare support implementation would help address some of these limitations. Similarly, additional studies correlating ITL or ITR to action would be helpful. It would also be ideal to study a wider range of institutions, including smaller organizations.

## Conclusions

The COVID-19 pandemic has had a myriad of effects on HCWs that put our workforce at risk. These data show an association between CCS and burnout, anxiety and depression, and ITL and ITR. Institutional interventions supporting childcare resources for HCWs may attenuate burnout, anxiety, depression, ITR, or ITL.

## Notes

Supplement. **eAppendix.** Survey Questions **eTable 1. **Multivariate (Logit) Models of Burnout Predictors Including Differential Effect (Interaction Term) of Gender and Childcare Stress **eTable 2.** Multivariate (Logit) Models of Predictors of Likelihood to Reduce Hours Including Differential Effect (Interaction Term) of Gender and Childcare Stress **eTable 3.** Multivariate (Logit) Models of Predictors of Likelihood to Leave Practice Including Differential Effect (Interaction Term) of Gender and Childcare Stress **eTable 4.** Multivariate (Logit) Models of Predictors of Anxiety and Depression Including Differential Effect (Interaction Term) of Gender and Childcare Stress
