# Supplementary material for: Childcare Stress, Burnout, and Intent to Reduce Hours or Leave the Job During the COVID-19 Pandemic Among US Health Care Workers
Source: JAMA Netw Open. 2022 Jul 18;5(7):e2221776. doi: 10.1001/jamanetworkopen.2022.21776 (PMC9294994; doi:10.1001/jamanetworkopen.2022.21776)
Supplement: Supplement. — eAppendix. Survey Questions eTable 1. Multivariate (Logit) Models of Burnout Predictors Including Differential Effect (Interaction Term) of Gender and Childcare Stress eTable 2. Multivariate (Logit) Models of Predictors of Likelihood to Reduce Hours Including Differential Effect (Interaction Term) of Gender and Childcare Stress eTable 3. Multivariate (Logit) Models of Predictors of Likelihood to Leave Practice Including Differential Effect (Interaction Term) of Gender and Childcare Stress eTable 4. Multivariate (Logit) Models of Predictors of Anxiety and Depression Including Differential Effect (Interaction Term) of Gender and Childcare Stress [file jamanetwopen-e2221776-s001.pdf]

Supplemental Online Content

Harry EM, Carlasare LE, Sinsky CA, et al. Childcare stress, burnout, and intent to reduce hours or leave the job during the COVID-19 pandemic among US health care workers. *JAMA Netw Open*. 2022;5(7):e2221776. doi:10.1001/jamanetworkopen.2022.21776

eAppendix. Survey Questions
eTable 1. Multivariate (Logit) Models of Burnout Predictors Including Differential Effect (Interaction Term) of Gender and Childcare Stress
eTable 2. Multivariate (Logit) Models of Predictors of Likelihood to Reduce Hours Including Differential Effect (Interaction Term) of Gender and Childcare Stress
eTable 3. Multivariate (Logit) Models of Predictors of Likelihood to Leave Practice Including Differential Effect (Interaction Term) of Gender and Childcare Stress
eTable 4. Multivariate (Logit) Models of Predictors of Anxiety and Depression Including Differential Effect (Interaction Term) of Gender and Childcare Stress

This supplemental material has been provided by the authors to give readers additional information about their work.

© 2022 Harry EM et al. *JAMA Network Open.*

eAppendix. Survey Questions

The stress I experienced today is

a. Minimal

b. Modest

c. High

d. Very high

I worry about exposing myself and my family to COVID

a. Not at all
b. Somewhat
c. Moderately
d. To a great extent

Due to the impact of COVID 19, I am experiencing the following:

a. Anxiety or depression [not at all, somewhat, moderately, to a great extent]

b. Work overload [not at all, somewhat, moderately, to a great extent]

c. Concerns about childcare [not at all, somewhat, moderately, to a great extent]

d. Worries that I have had to do things at work that compromise my integrity [not at all, somewhat, moderately, to a great extent]

Being part of the COVID-19 response has increased my sense of meaning and purpose

a. Not at all

b. Somewhat

c. Moderately

d. To a great extent

I feel valued by my organization

a. Not at all
b. Somewhat

c. Moderately

d. To a great extent

© 2022 Harry EM et al. *JAMA Network Open.*

Using your own definition of "burnout," please choose one of the answers below:
a. I enjoy my work. I have no symptoms of burnout
b. I am under stress, and don't always have as much energy as I did, but I don't feel burned out.
c. I am beginning to burn out and have one or more symptoms of burnout, e.g. emotional exhaustion
d. The symptoms of burnout that I am experiencing won't go away. I think about work frustrations a lot.
e. I feel completely burned out. I am at a point where I may need to seek help

Work Plans

What is the likelihood that you will reduce the number of hours you devote to clinical care over the next 12 months?
a. None
b. Slight
c. Moderate
d. Likely
e. Definitely

What is the likelihood that you would leave your practice within two years?
a. None
b. Slight
c. Moderate
d. Likely
e. Definitely

© 2022 Harry EM et al. *JAMA Network Open.*

eTable 1. Multivariate (Logit) Models of Burnout Predictors Including Differential Effect (Interaction Term) of Gender and Childcare Stress

	Model 1<br>Full Sample N = 35, 998				Model 2<br>Full Sample N = 35, 998			
	AOR<br>[95% CI]	p-value	ARR<br>[95% CI]	ARD<br>[95% CI]	AOR<br>[95% CI]	p-value	ARR<br>[95% CI]	ARD<br>[95% CI]
Sex								
Female	1.49<br>[1.41, 1.57]	< 0.001	1.22<br>[1.18, 1.25]	9.6%<br>[8.3, 10.8]	1.50<br>[1.42, 1.59]	<0.001	1.22<br>[1.18, 1.25]	9.6%<br>[8.3, 10.8]
Childcare risk								
High risk	1.80<br>[1.70, 1.90]	< 0.001	1.30<br>[1.27, 1.33]	14.2%<br>[12.9, 15.4]	1.86<br>[1.68, 2.06]	<0.001	1.30<br>[1.27, 1.33]	14.2%<br>[12.9, 15.4]
Sex X Childcare risk								
Female X High risk					0.95<br>[0.84, 1.07]	0.412	1.14<br>[1.09, 1.20]	8.46%<br>[5.9, 11.0]
Constant	0.39<br>[.26, .58]	<0.001			0.38<br>[.26, .57]	<0.001	ARR<br>[95% CI]	ARD<br>[95% CI]
MZ-R2	0.053				0.053			

AOR = adjusted odds ratio, ARR = adjusted risk ratio, ARD = adjusted risk difference, MZ-R2 = McKelvey and Zavoina's pseudo R2.

(a) = adjusted for specialty, years in practice, and role. (Interaction term) = females are 1.14 times more likely (14% more likely) to have higher burnout than males when they experience higher childcare stress, by 8.46%.

	Model 3<br>Physicians only<br>N = 12,888				Model 4<br>Physicians only<br>N = 12,888			
	AOR<br>[95% CI]	p-value	ARR<br>[95% CI]	ARD<br>[95% CI]	AOR<br>[95% CI]	p-value	ARR<br>[95% CI]	ARD<br>[95% CI]
Sex								
Female	1.54<br>[1.43, 1.67]	< 0.001	1.25<br>[1.20, 1.30]	10.4%<br>[8.6, 12.2]	1.56<br>[1.43, 1.70]	<0.001	1.25<br>[1.20, 1.30]	10.4%<br>[8.6, 12.2]
Childcare risk								

© 2022 Harry EM et al. *JAMA Network Open.*

High risk	1.92 [1.75, 2.10]	< 0.001	1.37 [1.32, 1.43]	15.8% [13.6, 17.9]	1.97 [1.73, 2.24]	<0.001	1.37 [1.32, 1.43]	15.8% [13.6, 17.9]
Sex X Childcare risk								
Female X High risk					0.95 [0.80, 1.13]	0.605	1.17 [1.10, 1.25]	9.5% [5.8, 13.2]
Constant	0.28 [.14, .56]	<0.001			0.28 [.14, .56]	<0.001		
MZ-R2	0.060				0.060			

AOR = adjusted odds ratio, ARR = adjusted risk ratio, ARD = adjusted risk difference, MZ-R2 = McKelvey and Zavoina's pseudo R2.

(a) = adjusted for specialty, years in practice, and role. (Interaction term) = females are 1.17 times more likely (17% more likely) to have higher burnout than males when they experience higher childcare stress, by 9.5%.

© 2022 Harry EM et al. *JAMA Network Open.*

eTable 2. Multivariate (Logit) Models of Predictors of Likelihood to Reduce Hours Including Differential Effect (Interaction Term) of Gender and Childcare Stress

	Model 1<br>Full Sample<br>N = 15,807				Model 2<br>Full Sample<br>N = 15,807			
	AOR<br>[95% CI]	p-value	ARR<br>[95% CI]	ARD<br>[95% CI]	AOR<br>[95% CI]	p-value	ARR<br>[95% CI]	ARD<br>[95% CI]
Sex								
Female	1.11<br>[1.02, 1.20]	< 0.001	1.07<br>[1.01, 1.13]	2.1%<br>[.45, 3.8]	1.06<br>[0.97, 1.16]	0.185	1.07<br>[1.01, 1.13]	2.1%<br>[.45, 3.8]
Childcare risk								
High risk	1.91<br>[1.76, 2.08]	< 0.001	1.52<br>[1.44, 1.60]	14.1%<br>[12.2, 16.0]	1.71<br>[1.48, 1.97]	<0.001	1.52<br>[1.44, 1.60]	14.1%<br>[12.2, 16.0]
Sex X Childcare risk								
Female X High risk					1.18<br>[1.00, 1.40]	0.050	1.06<br>[1.01, 1.12]	2.4%<br>[.52, 4.3]
Constant	0.17<br>[.08, .38]	<0.001			0.18<br>[.08, .39]	<0.001		
MZ-R2	0.059				0.055			

AOR = adjusted odds ratio, ARR = adjusted risk ratio, ARD = adjusted risk difference, MZ-R2 = McKelvey and Zavoina's pseudo R2.

(a) = adjusted for specialty, years in practice, and role. (Interaction term) = females are 1.06 times more likely (6% more likely) to have higher burnout than males when they experience higher childcare stress, by 2.4%.

	Model 3<br>Physicians only<br>N = 8,722				Model 4<br>Physicians only<br>N = 8,722			
	AOR<br>[95% CI]	p-value	ARR<br>[95% CI]	ARD<br>[95% CI]	AOR<br>[95% CI]	p-value	ARR<br>[95% CI]	ARD<br>[95% CI]
Sex								
Female	1.07	0.149	1.05	1.5%	1.01	0.841	1.05	1.5%

© 2022 Harry EM et al. *JAMA Network Open.*

	[0.97, 1.19]		[0.98, 1.12]	[-.55, 3.6]	[0.90, 1.13]		[0.98, 1.12]	[-.55, 3.6]
Childcare risk								
High risk	1.92 [1.71, 2.15]	<0.001	1.51 [1.41, 1.62]	14.1% [11.6, 16.7]	1.71 [1.46, 2.00]	<0.001	1.51 [1.41, 1.62]	14.1% [11.6, 16.7]
Sex X Childcare risk								
Female X High risk					1.26 [1.01, 1.56]	0.033	1.14 [1.03, 1.27]	5.7% [1.3, 10.1]
Constant	0.22 [.06, .72]	< 0.001			0.22 [.06, .72]	0.013		
MZ-R2	0.051				0.052			

AOR = adjusted odds ratio, ARR = adjusted risk ratio, ARD = adjusted risk difference, MZ-R2 = McKelvey and Zavoina's pseudo R2.

(a) = adjusted for specialty, and years in practice. (Interaction term) = females are 1.14 times more likely (14% more likely) to have higher burnout than males when they experience higher childcare stress, by 5.7%.

© 2022 Harry EM et al. *JAMA Network Open.*

eTable 3. Multivariate (Logit) Models of Predictors of Likelihood to Leave Practice Including Differential Effect (Interaction Term) of Gender and Childcare Stress

	Model 1<br>Full Sample<br>N = 15,997				Model 2<br>Full Sample<br>N = 15,997			
	AOR<br>[95% CI]	p-value	ARR<br>[95% CI]	ARD<br>[95% CI]	AOR<br>[95% CI]	p-value	ARR<br>[95% CI]	ARD<br>[95% CI]
Sex								
Female	0.97<br>[0.89, 1.06]	0.639	0.98<br>[0.92, 1.04]	.86%<br>[-0.20, 1.2]	0.95<br>[0.87, 1.05]	0.390	0.98<br>[0.92, 1.04]	-.38%<br>[-2.0, 1.3]
Childcare risk								
High risk	1.28<br>[1.17, 1.40]	<0.001	1.18<br>[1.12, 1.25]	4.9%<br>[3.2, 6.7]	1.20<br>[1.03, 1.40]	0.017	1.18<br>[1.12, 1.25]	4.8%<br>[3.0, 6.6]
Sex X Childcare risk								
Female X High risk					1.10<br>[0.917, 1.32]	0.302	1.03<br>[.92, 1.16]	1.0%<br>[-2.1, 4.2]
Constant	0.26<br>[.12, .54]	<0.001			0.26<br>[.13, .55]	<0.001		
MZ-R2	0.062				0.062			

AOR = adjusted odds ratio, ARR = adjusted risk ratio, ARD = adjusted risk difference, MZ-R2 = McKelvey and Zavoina's pseudo R2.
(b) = adjusted for specialty, years in practice, and role. (Interaction term) = females are 1.03 times more likely (3% more likely) to have higher burnout than males when they experience higher childcare stress, by 1.0%.

	Model 3<br>Physicians only<br>N = 8,743				Model 4<br>Physicians only<br>N = 8,743			
	AOR<br>[95% CI]	p-value	ARR<br>[95% CI]	ARD<br>[95% CI]	AOR<br>[95% CI]	p-value	ARR<br>[95% CI]	ARD<br>[95% CI]
Sex								
Female	0.97<br>[0.82, 1.09]	0.681	0.98	-.40<br>[-2.7, 1.4]	0.96<br>[0.84, 1.09]	0.587	0.98<br>[0.90, 1.06]	-.40<br>[-2.7, 1.4]

© 2022 Harry EM et al. *JAMA Network Open.*

			[0.90, 1.06]					
Childcare risk								
High risk	1.51 [1.32, 1.72]	<0.001	1.33 [1.22, 1.46]	7.2% [4.8, 9.6]	1.47 [1.23, 1.76]	<0.001	1.33 [1.22, 1.46]	7.2% [4.8, 9.6]
Sex X Childcare risk								
Female X High risk					1.04 [0.82, 1.34]	0.699	1.00 [0.87, 1.16]	.25% [-3.8, 4.3]
Constant	0.29 [.08, 1.02]	0.054			0.29 [.08, 1.02]	0.055		
MZ-R2	0.085				0.086			

AOR = adjusted odds ratio, ARR = adjusted risk ratio, ARD = adjusted risk difference, MZ-R2 = McKelvey and Zavoina's pseudo R2.

(a) = adjusted for specialty, years in practice, and role. (Interaction term) = females are 1.00 times more likely (0% more likely) to have higher burnout than males when they experience higher childcare stress, by 0.25%.

© 2022 Harry EM et al. *JAMA Network Open.*

eTable 4. Multivariate (Logit) Models of Predictors of Anxiety and Depression Including Differential Effect (Interaction Term) of Gender and Childcare Stress

	Model 1<br>Full Sample<br>N = 39,757				Model 2<br>Full Sample<br>N = 39,757			
	AOR<br>[95% CI]	p-value	ARR<br>[95% CI]	ARD<br>[95% CI]	AOR<br>[95% CI]	p-value	ARR<br>[95% CI]	ARD<br>[95% CI]
Sex								
Female	1.56<br>[1.47, 1.65]	< 0.001	1.33<br>[1.28, 1.38]	9.2%<br>[8.1, 10.4]	1.62<br>[1.52, 1.73]	<0.001	1.33<br>[1.28, 1.38]	9.2%<br>[8.1, 10.4]
Childcare risk								
High risk	2.15<br>[2.04, 2.26]	< 0.001	1.55<br>[1.51, 1.59]	17.2%<br>[16.0, 18.4]	2.43<br>[2.19, 2.70]	<0.001	1.55<br>[1.51, 1.59]	17.2%<br>[16.0, 18.4]
Sex X Childcare risk								
Female X High risk					0.85<br>[0.75, 0.95]	0.007	1.18<br>[1.11, 1.24]	7.9%<br>[5.4, 10.4]
Constant	0.17<br>[.11, .25]	< 0.001			0.16<br>[.11, .25]	<0.001		
MZ-R2	0.096				0.097			

AOR = adjusted odds ratio, ARR = adjusted risk ratio, ARD = adjusted risk difference, MZ-R2 = McKelvey and Zavoina's pseudo R2.

(a) = adjusted for specialty, years in practice, and role. (Interaction term) = females are 1.18 times more likely (18% more likely) to have higher burnout than males when they experience higher childcare stress, by 7.9%.

	Model 3<br>Physicians only<br>N = 14,194				Model 4<br>Physicians only<br>N = 14,194			
	AOR<br>[95% CI]	p-value	ARR<br>[95% CI]	ARD<br>[95% CI]	AOR<br>[95% CI]	p-value	ARR<br>[95% CI]	ARD<br>[95% CI]
Sex								
Female	1.55	<0.001	1.37	8.0%	1.68	<0.001	1.37	8.0%

© 2022 Harry EM et al. *JAMA Network Open.*

	[1.43, 1.69]		[1.29, 1.46]	[6.5, 9.6]	[1.53, 1.85]		[1.29, 1.46]	[6.5, 9.6]
Childcare risk								
High risk	2.11 [1.92, 2.31]	<0.001	1.68 [1.58, 1.79]	14.8% [12.9, 16.8]	2.47 [2.16, 2.83]	<0.001	1.68 [1.58, 1.79]	14.8% [12.9, 16.8]
Sex X Childcare risk								
Female X High risk					0.75 [.63, .89]	0.001	1.15 [1.05, 1.27]	5.5% [2.0, 9.0]
Constant	0.26 [.13, .53]	<0.001			0.25 [.12, .52]	<0.001		
MZ-R2	0.062				0.064			

AOR = adjusted odds ratio, ARR = adjusted risk ratio, ARD = adjusted risk difference, MZ-R2 = McKelvey and Zavoina's pseudo R2.

(a) = adjusted for specialty, and years in practice. (Interaction term) = females are 1.15 times more likely (15% more likely) to have higher anxiety/depression than males when they experience higher childcare stress, by 5.5%.

© 2022 Harry EM et al. *JAMA Network Open.*
